# Supplementary material for: Clinical Characteristics of Patients with SARS-CoV-2 N501Y Variants in General Practitioner Clinic in Japan
Source: J Clin Med. 2021 Dec 14;10(24):5865. doi: 10.3390/jcm10245865 (PMC8708988; doi:10.3390/jcm10245865)
Supplement: Supplementary file 1 [file jcm-10-05865-s001.zip › jcm-1435770-supplementary.pdf]

Table S1. STROBE Statement—Checklist of items that should be included in reports of **cohort studies**

	Item No	Recommendation	
**Title and abstract**	1	(*a*) Indicate the study's design with a commonly used term in the title or the abstract	Abstract (p 1)
		(*b*) Provide in the abstract an informative and balanced summary of what was done and what was found	Abstract (p 1)
**Introduction**			
Background/rationale	2	Explain the scientific background and rationale for the investigation being reported	1.Introduction (p 1-2)
Objectives	3	State specific objectives, including any prespecified hypotheses	1.Introduction (p 2)
**Methods**			
Study design	4	Present key elements of study design early in the paper	2.1. Data source and study design (p 2-3)
Setting	5	Describe the setting, locations, and relevant dates, including periods of recruitment, exposure, follow-up, and data collection	2.1. Data source and study design (p 2-3)
Participants	6	(*a*) Give the eligibility criteria, and the sources and methods of selection of participants. Describe methods of follow-up	2.1. Data source and study design (p 2-3)
		(*b*) For matched studies, give matching criteria and number of exposed and unexposed	(N/A)
Variables	7	Clearly define all outcomes, exposures, predictors, potential confounders, and effect modifiers. Give diagnostic criteria, if applicable	2.1. Data source and study design(p 2-3) 2.3. Screening of N501Y mutation (p 4) 2.4. Evaluation of CT scan (p 4)
Data sources/ measurement	8*	For each variable of interest, give sources of data and details of methods of assessment (measurement). Describe comparability of assessment methods if there is more than one group	2.5. Statistics (p 4)
Bias	9	Describe any efforts to address potential sources of bias	2.5. Statistics (p 4) 4. Discussion (p 11-12)

Study size	10	Explain how the study size was arrived at	2.1. Data source and study design (p 2)
Quantitative variables	11	Explain how quantitative variables were handled in the analyses. If applicable, describe which groupings were chosen and why	2.5. Statistics (p 4)
Statistical methods	12	(*a*) Describe all statistical methods, including those used to control for confounding	2.5. Statistics (p 4)
		(*b*) Describe any methods used to examine subgroups and interactions	(N/A)
		(*c*) Explain how missing data were addressed	2.1. Data source and study design (p 3)
		(*d*) If applicable, explain how loss to follow-up was addressed	(N/A)
		(*e*) Describe any sensitivity analyses	(N/A)
**Results**			
Participants	13*	(a) Report numbers of individuals at each stage of study—eg numbers potentially eligible, examined for eligibility, confirmed eligible, included in the study, completing follow-up, and analysed	3. Results (p 5-11)
		(b) Give reasons for non-participation at each stage	3.1. Characteristics (p 5)
		(c) Consider use of a flow diagram	(N/A)
Descriptive data	14*	(a) Give characteristics of study participants (eg demographic, clinical, social) and information on exposures and potential confounders	3.1. Characteristics (p 5)
		(b) Indicate number of participants with missing data for each variable of interest	Table 1. (p 5-6)
		(c) Summarise follow-up time (eg, average and total amount)	3.2. Clinical symptoms (p 8)
Outcome data	15*	Report numbers of outcome events or summary measures over time	3. Results (p 5-11)
Main results	16	(*a*) Give unadjusted estimates and, if applicable, confounder-adjusted estimates and their precision (eg, 95% confidence interval). Make clear which confounders were adjusted for and why they were included	Table 4. (p 9)
		(*b*) Report category boundaries when continuous variables were categorized	Table 1. (p 5-6) Table S4.

			2.1. Data source and study design (p 3)
		(*c*) If relevant, consider translating estimates of relative risk into absolute risk for a meaningful time period	(N/A)
Other analyses	17	Report other analyses done—eg analyses of subgroups and interactions, and sensitivity analyses	(N/A)
**Discussion**			
Key results	18	Summarise key results with reference to study objectives	4. Discussion (p 11)
Limitations	19	Discuss limitations of the study, taking into account sources of potential bias or imprecision. Discuss both direction and magnitude of any potential bias	4. Discussion (p 12)
Interpretation	20	Give a cautious overall interpretation of results considering objectives, limitations, multiplicity of analyses, results from similar studies, and other relevant evidence	4. Discussion (p 11-12)
Generalisability	21	Discuss the generalisability (external validity) of the study results	4. Discussion (p 12)
**Other information**			
Funding	22	Give the source of funding and the role of the funders for the present study and, if applicable, for the original study on which the present article is based	Funding (p 13)

Table S2. Details of the exposure settings of the patients infected with wild-type 501N and N501YV. (n = 52).

	Total n = 52		Wild-type 501N n = 28		N501YV n = 24		p-Value
Social gatherings, n (%)	9	(17.3)	3	(10.7)	6	(25.0)	0.061
Household, n (%)	6	(11.5)	6	(21.4)	0	(0)	
Healthcare, n (%)	2	(3.9)	2	(7.1)	0	(0)	
Workplace (non-Healthcare), n (%)	9	(17.3)	5	(17.9)	4	(16.7)	
Unknown, n (%)	26	(50.0)	12	(42.9)	14	(58.3)	

Table S3. Prescribed drugs for the patients infected with wild-type 501N and N501YV. (n = 52).

	Total n = 52		Wild-type 501N n = 28		N501YV n = 24		p-Value
Drugs							
Antitussives, n (%)	6	(11.5)	4	(14.3)	2	(8.3)	0.503
Inhaled corticosteroids, n (%)	5	(9.6)	3	(10.7)	2	(8.3)	0.772
Antibiotics, n (%)	6	(11.5)	4	(14.3)	2	(8.3)	0.503

Table S4. Baseline characteristics of patients who were directly hospitalized and those who stayed in designated non-health facilities before hospitalization.

	**Total n = 52**		**Directly hospitalized n = 47**		**Pre-hospital isolation n = 5**		**p-Value**
Median age (years)	32.5	IQR 23.5-49	30	IQR 23-48	48	IQR 40-51	0.103
Average age (years)	37.4	±16.1	36.5	±16.6	46.2	±7.5	0.203
Sex							
Male, n (%)	29	(55.8)	27	(57.5)	2	(40.0)	0.455
Female, n (%)	23	(44.2)	20	(42.6)	3	(60.0)	
BMI							
Under weight (<18.5), n (%)	5	(9.6)	5	(10.6)	0	(0)	0.209
Normal weight (18.5-23.9), n (%)	25	(48.1)	24	(51.1)	1	(20.0)	
Overweight (24.0-27.9), n (%)	13	(25.0)	11	(23.4)	2	(40.0)	
Obesity (28+), n (%)	7	(13.5)	6	(12.8)	1	(20.0)	
Missing, n (%)	2	(3.9)	1	(2.1)	1	(20.0)	
Smoking status (current)							
No, n (%)	38	(73.1)	33	(70.2)	5	(100)	0.361
Yes, n (%)	12	(23.1)	12	(25.5)	0	(0)	
Missing, n (%)	2	(3.9)	2	(4.3)	0	(0)	
High-risk medical condition for severe COVID-19							
Yes, n (%)	11	(21.2)	10	(21.3)	1	(20.0)	0.947
Birth country							
Japan, n (%)	37	(71.2)	34	(72.3)	3	(60.0)	0.563
Other country, n (%)	15	(28.9)	13	(27.7)	2	(40.0)	
Unknown transmission route							
Yes, n (%)	26	(50.0)	23	(48.9)	3	(60.0)	0.638
Pre-treatment symptoms							
Changes in appetite, n (%)	10	(19.2)	6	(12.8)	4	(80.0)	**<0.001**
Shortness of breath, n (%)	6	(11.5)	3	(6.4)	3	(60.0)	**<0.001**
Febrile over 38°C, n (%)	27	(51.9)	22	(46.8)	5	(100)	**0.024**
Average resting oxygen saturation	98.8	±1.0	98.9	±1.0	98.2	±0.8	0.155
	Total* n = 43		Directly hospitalized n = 38		Pre-hospital isolation n = 5		
Average period of onset to admission (days)	3.7	±2.3	3.5	±2.3	5.8	±1.3	**0.031**

	Total** N= 51		Directly hospitalized N= 46		Pre-hospital isolation N= 5		
Average CT severity score	3.1	±3.4	2.5	±2.9	9.0	±1.6	**<0.001**

* Nine patients who were asymptomatic at the time of COVID-19 diagnostic test which was conducted for contact tracing were excluded. ** One patient who did not undergo CT scan was excluded.
